# Distance in Distant Care: Qualitative Content Analysis of Providers’ Experiences in Tele–Mental Care

**DOI:** 10.2196/38568

**Published:** 2023-02-17

**Authors:** Jill W Åhs, Albertine Ranheim, Erica Mattelin, Henrik Eriksson, Monir Mazaheri

**Affiliations:** 1 Department of Health Sciences Swedish Red Cross University Huddinge Sweden; 2 Division of Nursing Department of Neurobiology, Care Sciences, and Society Karolinska Institute Huddinge Sweden; 3 Center for Social and Affective Neuroscience Department of Biomedical and Clinical Sciences Linköping University Linköping Sweden; 4 Barnafrid, Swedish National Center on Violence Against Children Department of Biomedical and Clinical Sciences Linköping University Linköping Sweden; 5 Section for Health Promotion and Care Sciences University West Trollhättan Sweden; 6 Department of Nursing Sciences Sophiahemmet University Stockholm Sweden

**Keywords:** telehealth, telemedicine, patient care, mental health, care delivery, communication technology, patient-provider, provider, provider experience, health care professional, experience, content analysis, qualitative

## Introduction

Tele–mental care is ubiquitous worldwide [1]. It is effective and even preferred by patients [2]. Yet, providers have expressed concerns that the technology limits conveying nonverbal cues [3,4] or impedes the therapeutic relationship [3,5]. These key aspects of communication and interpersonal connection in care could be described as types of distances, where spatial distance could prevent patient observation, and psychological distance may prevent effective interpersonal connection. Considering the growing use of tele–mental care and the relevance of these forms of distance for successful tele–mental care encounters, this study was conducted to explore these or other forms of “distance” that arise in providers’ descriptions of tele–mental care encounters.

## Methods

### Overview

Study information was shared with clinic heads and providers working in public and private clinics in Sweden. In-depth interviews (range 45-75 minutes) were conducted with 7 providers, including doctors, psychologists, and a social worker, all with tele–mental care experience. Directed content analysis [6] was performed secondarily, whereby meaning units relating to latent or literal “distance” were coded, categorized, and clustered into themes. Themes are presented below with example data.

### Ethics Approval

This research adhered to the Declaration of Helsinki, ensuring informed consent, confidentiality, and the rights of participants, and was approved by the Swedish Ethical Review Authority (Dnr 2019-06412).

## Results

Providers described different forms of “distance” that are present in tele–mental care encounters. Two juxtaposing themes are presented in this brief report to describe hindrances or enablers of successful tele–mental care.

### Distance Was Magnified in the Distant Encounter

Providers described how they and their colleagues who rapidly switched to telecare for COVID-19 mitigation were distant to the idea of providing remote patient care. Providers had to overcome psychological distance or be accepting of the idea. “So many of us had huge problems with even...to mentally get close to the idea of having conversations over the internet with patients” (P4).

The distance was found to be exaggerated in meetings with certain patients and patient groups. “Different people respond in different ways” (P7). Tele–mental care was described as unsuitable for the patient who does not feel secure or trust confidentiality in the technology-mediated encounter, or for those who struggle with expressing or processing others’ emotions. Patients may seem distant or harder to establish rapport with through telecare.

### Distance Was Bridged in the Distant Encounter

In removing the physical distance to travel to the clinic, providers described the benefits of telecare for patients who “would avoid to go out” (P5) or who “...wouldn't step inside my office no matter what” (P6). These patients could now gain contact with the provider to begin “...building a relationship...gradually...from home...to reach the life outside” (P3). Tele–mental care is a bridge to accessing care for patients who have challenges with traveling or the social demands or busy atmosphere of the clinic. As distractions are removed between the patient and provider, the patient is afforded the necessary focus.

Providers described that the patient could “get more to the point” (P2) in tele–mental care. The modality shielded patients from the vulnerability of sharing emotions. The spatial distance reduced patients’ apprehensions to share thoughts they held close.

For providers, technology could bridge the spatial distance from the patient. Cameras in video-based care could allow for closer observation of patients’ facial expressions. Seeing facial expressions more clearly made it easier for providers to read the emotions of the patient “...because you get really up close” (P2).

## Discussion

Providers described different forms of distance in tele–mental care. Spatial distance could bring with it the positive impacts of reducing emotional apprehensions for patients and providing “a safe distance” for patients to reveal their feelings [7]. For certain patients or patient groups though, telecare was found to be unsuitable, which has been noted previously [3]. As in previous research, providers in this study expressed concerns about adopting telecare practices [3,5]. Yet, they also found it connected them with patients who would otherwise not travel to meet with them [7].

Readers should consider that the findings are drawn from interviews with few providers and may vary from the experiences of other providers. The study does not purport generalizable findings but explores examples of “distance” described by providers in telecare. These findings may be useful for care providers or clinic managers to improve awareness of the roles technology may have in impacting communications and interpersonal connection in tele–mental care [8]. Approaches to reduce or resolve the negative impacts of distance in tele–mental care may better allow therapeutic goals to be attained.
